# Endovascular therapy in basilar artery occlusion in Sweden 2016–2019—a nationwide, prospective registry study

**DOI:** 10.1007/s00234-021-02843-3

**Published:** 2021-10-30

**Authors:** Birgitta Ramgren, Petrea Frid, Bo Norrving, Johan Wassélius, Teresa Ullberg

**Affiliations:** 1 Diagnostic Radiology, Department of Clinical Sciences Lund, Lund University, Lund, Sweden; 2 Section of Neuroradiology and Odontology, Center for Medical Imaging and Physiology, Skåne University Hospital, 221 85 Lund, Sweden; 3 Neurology, Department of Clinical Sciences Lund, Lund University, Lund, Sweden; 4 Neurology, Skåne University Hospital, Lund/Malmö, Sweden

**Keywords:** Acute stroke, Endovascular recanalization, Thrombectomy, Posterior circulation, Outcome, Basilar artery occlusion

## Abstract

**Purpose:**

We present the first nationwide study on endovascular therapy for basilar artery occlusion (BAO) from early hospital management to 3-month outcome.

**Methods:**

Data were collected on all acute ischaemic stroke patients registered 2016–2019 in the two national quality registers for stroke care and endovascular therapy (EVT), receiving EVT for BAO and subclassified into proximal, middle and distal.

**Results:**

In all, 251 patients were included: 69 proximal, 73 middle and 109 distal BAO. Patients with proximal BAO were younger (66, middle 71, distal 76, *p* < 0.0001), less often female (27.5%, middle 47.9%, distal 47.7%, *p* = 0.015), more often smokers (28.6%, middle 20.3%, distal 11.5%, *p* < 0.0001), and fewer had atrial fibrillation (13.2%, middle 24.7%, distal 48.6%, *p* < 0.0001). Level of consciousness and NIHSS score did not differ by BAO subtype and 52.2% were alert on admission. Time from groin puncture to revascularization was significantly longer in patients with proximal BAO (71, middle 46, distal 42 min, *p* < 0.0001), and angioplasty and/or stenting was more often performed in patients with proximal (43.4%) and middle (27.4%) than distal (6.4%) BAO (*p* < 0.0001). Cumulative 90-day mortality was 38.6% (proximal 50.7%, middle 32.9%, distal 34.9%, *p* = 0.02). Older and pre-stroke dependent patients had higher mortality, as did patients in whom angioplasty/stenting was performed.

**Conclusion:**

We confirm a serious outcome in BAO despite endovascular therapies, and demonstrate important differences relating to occlusion location in baseline characteristics, procedural time, therapeutic measures and outcome. Further in-depth analyses of factors affecting outcome in BAO are warranted.

**Supplementary Information:**

The online version contains supplementary material available at 10.1007/s00234-021-02843-3.

## Introduction

Ischaemic stroke caused by large vessel occlusion (LVO) in the vertebrobasilar arteries is a rare, but devastating, and often fatal subtype of occlusive cerebrovascular disease. Its natural course and outcome without treatment are generally regarded as dismal. For patients with basilar artery occlusion (BAO) not receiving reperfusion treatment, reported mortality rates in the immediate post-stroke period (0–3 months) vary between 40 and 86% [1, 2]. The introduction of reperfusion therapies such as intravenous (IV) or intra-arterial thrombolysis has not significantly altered mortality rates in this patient group [3, 4].

Endovascular thrombectomy (EVT) for LVO in the anterior circulation has become standard treatment in the wake of a series of successful randomized clinical trials [5–9] and meta-analyses [10, 11] showing superior outcome for patients treated with EVT vs conventional treatment.

In contrast, only a handful of registry studies [12–15], single- [16–23] and multicentre studies [24, 25], meta-analysis [26] and randomized clinical trials (RCTs) [27, 28] have been published on endovascular treatment benefits and outcome in patients with BAO. The BEST RCT, which was stopped early due to poor recruitment and high crossover rate, did not show superiority of EVT over IVT [27]. The newly published BASICS RCT was also neutral, although 29.9% of eligible patients were not randomized, which may have introduced selection bias, and 79% of non-randomized patients underwent EVT outside the trial [28]. Thus, evidence is still unclear on the potential benefits of EVT for BAO [29]. In fact, RCTs for BAO have been questioned in part on the grounds that they may result in treatment being withheld from patients who could potentially benefit, for being vulnerable to selection bias, and that the principles of EVT benefit have already been proven in RCTs in the anterior circulation [30]. Views favouring RCTs emphasize the fact that differences in vascular anatomy between the anterior and posterior circulation may influence treatment response, making extrapolation of results from RCTs of the anterior circulation tenuous. Moreover, IVT for BAO may be more effective due to collateral flow, resulting in therapeutic effect on both ends of the thrombus. Lastly, observational studies showing superiority of EVT over IVT may also be biased (confounding by indication) [31]. Despite the limited RCT data, Swedish [32], European [33] and American [34] stroke guidelines recommend EVT in selected patients with BAO.

Combining data from two prospective registries with nationwide coverage, the present study aims to provide a comprehensive description of clinical characteristics, stroke workflow, treatment and outcome in patients receiving EVT for BAO, with stratification by lesion location.

## Methods

### Patient database

All patients > 18 years registered in both the Swedish Stroke Register (RS) and the Endovascular Treatment of Acute Stroke Register (EVAS) during 2016–2019 with LVO in the basilar artery were included.

### Data sources

#### The Swedish Stroke Register

Since 1994, RS [35] has served as the Swedish quality register for stroke care. All Swedish hospitals managing acute stroke (currently 72 sites) contribute to the data collection, and 90% of hospitalized stroke patients are included. Data collected in RS included demographics, pre-stroke function, vascular risk factors, stroke care and treatment. At 3 months post-stroke, patients are followed up by postal questionnaires, telephone interviews or in-person hospital visits, and variables are collected on living conditions, functional outcome, need of help and support, post-stroke problems and unmet needs. Mortality status and date of death are continuously obtained from the Swedish Causes of Death Register, a register governed by the Swedish Board of Health and Welfare, with > 99% coverage.

#### The Swedish Endovascular Treatment of Acute Stroke Register

Since 2014, EVAS [36] has served as the Swedish quality register for endovascular therapy in acute ischaemic stroke performed at all six comprehensive stroke centres (CSCs) in Sweden. The coverage has improved and has reached > 90%. Detailed data are collected on pre- and post-treatment radiology findings, procedural, technical and early clinical outcome data.

#### The merged RSEVAS database

In 2020, data on all acute ischaemic stroke patients treated with EVT for BAO and registered in RS and EVAS were merged into RSEVAS, using the Swedish personal identification number as key [37]. The result was a national database including detailed variables on pre-, peri- and post-stroke care for EVTs performed in Sweden 2016–2019.

### Main variables

#### BAO definition

BAO was defined based on digital subtraction angiography (DSA) and subdivided into three groups: proximal, middle and distal BAO [38]. Lesion location was defined by the most proximal segment of the BAO involved.

#### Baseline and stroke characteristics and workflow

Demographic characteristics from RS included age and sex, pre-stroke function and vascular risk factors. Functional status in RS is assessed using variables on dressing, toileting, mobility, living situation and need of help or support, and translated into modified Rankin Scale (mRS) using a previously validated algorithm with good agreement with objectively assessed 3-month mRS [39]. Clinical baseline characteristics included the National Institutes of Health Stroke Scale (NIHSS) score and level of consciousness (LOC) based on the Reaction Level Scale (RLS-85), which is widely used in Sweden. The RLS-85 is an eight-point grading scale with three main categories of alert, drowsy and comatose, which correlates well with the internationally used Glasgow Coma Scale [40]. Treatment data such as ongoing anticoagulation, IVT, process times such as onset to needle (OTN), door to needle (DTN), onset to groin puncture (OTG), onset to revascularization (OTR) and groin to revascularization time were registered. Time of onset was noted as unknown, known or estimated; known or estimated were registered as exact timepoints (YYMMDD hh:mm) and therefore grouped. Mode of patient transfer was defined as either secondary transport from a primary stroke centre or directly to a CSC.

#### Radiology and EVT data

Pre- and post-stroke imaging modality was registered. Technical variables such as use of conscious sedation (CS); general anaesthesia (GA) and access used, i.e. femoral, brachial or radial artery, were noted. Treatment strategies for thrombus removal such as aspiration alone or stent retriever and aspiration in combination were noted as was the use of angioplasty, stenting or both. Degree of successful reperfusion was defined as 2b–3 using the modified thrombolysis in cerebral infarction (mTICI) [41] scale ranging from 0 to 3. Brain imaging was performed within 24 h after EVT to detect possible ischaemia or haemorrhage.

#### Outcome variables

Early outcome was defined as NIHSS score at 24 h. Mortality within 24 h and at 7 and 28 days was noted. Any serious complications, grouped as procedure-related vs postprocedural, were registered by the treating neurologist and neurointerventionalist and are shown in detail in supplemental Table I a–d. Symptomatic intracerebral haemorrhage (sICH) was defined as presence of any haemorrhage with deterioration in NIHSS score by ≥ 4 points or death. Outcome at 90 days was estimated using the mRS 0–2, 3, 4, 5 and death.

### Missing data

Missing data included missing or incomplete data for individual variables in registered patients, as well as patients not returning the follow-up questionnaire.

### Statistics

IBM SPSS Statistics version 25 was used for all statistical analyses. Categorical variables were summarized as proportions and compared with Pearson’s χ^2^ test. Medians were compared using Kruskal–Wallis test. Means were compared using ANOVA test. Kaplan–Meier life tables were used to calculate probability of survival as a function of time, and results were displayed as survival curves. Log rank test was used to compare groups.

Functional outcome at 90 days is estimated using the mRS. In patients alive but lost to 90-day follow-up, results are shown both including missing data and using omission from analysis with extrapolation of functional outcome data from followed up survivors.

## Results

### Baseline characteristics

We included 251 patients with BAO on DSA subclassified into proximal (*n* = 69), middle (*n* = 73) and distal (*n* = 109). Baseline characteristics are presented in Table 1. Patients with proximal BAO were younger (66, middle 71, distal 76, *p* < 0.0001), less often female (proximal 27.5%, middle 47.9%, distal 47.7%, *p* = 0.015), more often smokers (proximal 28.6%, middle 20.3%, distal 11.5%, *p* < 0.0001), and fewer had atrial fibrillation (proximal 13.2%, middle 24.7%, distal 48.6%, *p* < 0.0001). NIHSS score was registered in 193 patients (77%), and median or mean NIHSS scores did not differ by occlusion location. LOC upon admission was registered in 99%, and approximately half were alert.

**Table 1:** Baseline characteristics in 251 patients with basilar artery occlusion (BAO). Data were missing for smoking (22.3%), pre-stroke dependency (5.6%), NIHSS (23.1%) and IVT (4%). The remaining variables had < 1.5% missing data

Variable	Proximal BAO *n* = 69 % (*n*)	Mid BAO *n* = 73 % (*n*)	Distal BAO *n* = 109 % (*n*)	All BAO *n* = 251 % (*n*)	*p* value
Demographics					
Median age (IQR)	66 (58–76)	71 (59–78)	76 (66–82)	72 (61–80)	< 0.001
Female sex	27.5% (19)	47.9% (35)	47.7% (52)	42.2% (106)	0.015
Pre-stroke function					0.419
mRS 0–2	92.3% (60)	88.2% (60)	85.6% (89)	88.2% (209)	
mRS 3–5	7.7% (5)	11.8% (8)	14.4% (15)	11.8% (28)	
Vascular risk factors					
Hypertension	60.9% (42)	56.2% (41)	68.8% (75)	62.9% (158)	0.393
AF total	13.2% (9)	24.7% (18)	48.6% (53)	32% (80)	< 0.001
Diabetes	20.3% (14)	16.4% (12)	22.0% (24)	19.9% (50)	0.642
Current smoking	28.6% (14)	20.3% (12)	11.5% (10)	18.5% (36)	0.043
Previous stroke	14.5% (10)	12.3% (9)	15.6% (17)	14.3% (36)	0.260
Previous TIA	5.8% (4)	4.1% (3)	4.6% (5)	4.8% (12)	0.573
Clinical characteristics					
NIHSS score (IQR)					
Median (IQR)	12 (6–26)	15 (7–30)	12 (6–28)	14 (6–29)	0.620
Mean (SD)	16 (10.8)	17 (10.2)	16 (11.0)	16 (10.6)	0.764
LOC					0.319
Alert	61.8% (42)	50.7% (37)	47.2% (51)	52.2% (130)	
Drowsy	17.6% (12)	26% (19)	22.4% (24)	22.1% (55)	
Comatose	20.6% (14)	23.3% (17)	30.6% (33)	25.7% (64)	
Treatments					
Ongoing OAC	2.9% (2)	9.6% (7)	22% (24)	13.1% (33)	0.001
IVT	29.2% (19)	43.1% (31)	31.7% (33)	34.4% (83)	0.175
OTN time (min, IQR)	143 (112–178)	137 (97–180)	115 (97–148)	124 (104–178)	0.134
DTN time (min, IQR)	67 (38–94)	46 (29–76)	64 (41–88)	60 (37–81)	0.183
Time of onset					0.702
Unknown	17.4% (12)	13.7% (10)	18.3% (20)	16.7% (42)	
Known or estimated	82.6% (57)	86.3% (63)	81.7% (89)	83.3% (209)	

*BAO* basilar artery occlusion, *IQR* interquartile range, *SD* standard deviation, *mRS* modified Rankin Scale, *TIA* transient ischemic attack, *NIHSS* National Institutes of Health Stroke Scale, *IVT* intravenous thrombolysis, *OTN* onset to needle, *DTN* door to needle, *OAC* oral anticoagulant, *LOC* level of consciousness

The majority (82.9%) had a known or estimated time of symptom onset. One-third received IVT; OTN and DTN times did not differ by BAO subtype.

### Mode of patient triage and stroke imaging

**Fig. 1:**
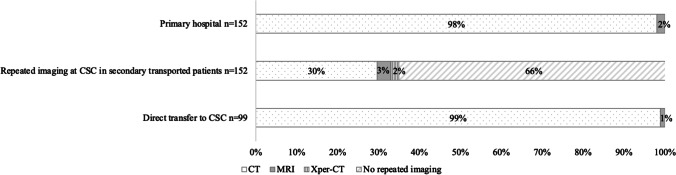
Imaging modality in patients transferred from a primary stroke centre (*n* = 152) and those (*n* = 99) transferred directly to a comprehensive stroke centre (CSC). Thirty-five percent of secondary transported patients had repeated imaging upon arrival to the CSC

A total of 152 (60.6%) patients arrived at a primary stroke centre and were subsequently referred to a CSC, while the remaining 99 (39.4%) were transported directly to a CSC. Stroke parenchymal and vascular imaging was performed using computed tomography (CT) in the vast majority of patients (Fig. 1). Of those arriving with secondary transport, imaging was repeated on arrival to the CSC in approximately one-third, CT-based for the majority (Fig. 1).

### Treatment strategies and process time

Just over half of patients had groin puncture within 6 h of symptom onset (Table 2). Median OTG time was 285 (185–421) min (4.7 h). Median time from OTG was significantly longer in patients with secondary transport (316 vs 195 min, *p* < 0.0001), as was median time from OTR (402 vs 248 min, *p* < 0.0001). Time from groin puncture to revascularization was significantly longer in proximal BAO (71, middle 46, distal 42 min, *p* < 0.0001). Aspiration only was used in 39.4% of cases, and stent retriever with or without aspiration was used in 53.4% of cases, with no differences in relation to occlusion location. Successful recanalization was achieved in 83.1%, with lower recanalization rates in proximal occlusions (75%), although the difference did not reach statistical significance. Angioplasty and/or stenting was more often used in proximal (43.4%) and middle (27.4%) than distal (6.4%) BAO (*p* < 0.0001). Cases with angioplasty or stenting (*n* = 57) were treated with periprocedural i.v. acetylsalicylic acid in 35.1% of cases, with glycoprotein IIb/IIIa inhibitors in 29.8% of cases and with both in 4% of cases.

**Table 2:** Triage, treatment strategies and process times. Missing data: unknown time of onset 16.7%, < 1.5% for the remaining variables

Variable	Proximal BAO *n* = 69 % (*n*)	Mid BAO *n* = 73 % (*n*)	Distal BAO *n* = 109 % (*n*)	All BAO *n* = 251 % (*n*)	*p* value
Mode of triage					0.052
Secondary transport	69.6% (48)	64.4% (47)	52.3% (57)	60.6% (152)	
Direct transport	30.4% (21)	35.6% (26)	47.7% (52)	39.4% (99)	
Process time (min, IQR)					
Groin puncture < 6 h	53.6% (37)	57.5% (42)	58.7% (64)	57% (143)	0.794
Onset to groin puncture	314 (208–447)	301 (200–425)	265 (165–402)	285 (185–421)	0.112
Onset to revascularization	409 (258–524)	366 (251–516)	314 (213–462)	360 (243–502)	0.054
Groin to revascularization	71 (45–120)	46 (23–86)	42 (24–71)	50 (26–91)	< 0.001
Anaesthesia					0.103
General	85.5% (59)	78.1% (57)	75.2% (82)	78.9% (198)	
Conscious sedation	14.5% (10)	19.2% (14)	24.8% (27)	20.3% (51)	
Converted to general	0% (0)	2.7% (2)	0% (0)	0.8% (2)	
Access					0.488
Femoral	91.3% (63)	95.8% (69)	90.8% (99)	92.4% (231)	
Brachial	2.9% (2)	1.4% (1)	5.5% (6)	3.6% (9)	
Radial	0% (0)	1.4% (1)	0.9% (1)	0.8% (2)	
Converted	5.8% (4)	1.4% (1)	2.8% (3)	3.2% (8)	
Treatment strategy					0.162
Aspiration alone	26.1% (18)	43.8% (32)	45.0% (49)	39.4% (99)	
Stent retriever (+ -aspiration)	63.8% (44)	50.7% (37)	48.6% (53)	53.4% (134)	
Only attempted EVT	5.8% (4)	4.1% (3)	5.5% (6)	5.2% (13)	
Other	4.3% (3)	1.4% (1)	0.9% (1)	2% (5)	
Angioplasty/stenting					< 0.001
Angioplasty alone	15.9% (11)	8.2% (6)	2.8% (3)	8% (20)	
Stenting alone	13.0% (9)	8.2% (6)	2.8% (3)	7.2% (18)	
Angioplasty + stenting	14.5% (10)	11% (8)	0.9% (1)	7.6% (19)	
Degree of revascularization					
Successful (mTICI 2b–3)	75.0% (51)	84.9% (62)	87.0% (94)	83.1% (207)	0.103

*BAO* basilar artery occlusion, *IQR* interquartile range, *EVT* endovascular therapy, *mTICI* modified thrombolysis in cerebral infarction

### Early postprocedural clinical and safety outcomes

Nine patients (3.6%) died within 24 h (Table 3). NIHSS score at 24 h could be evaluated in 60.7% (147/242) of survivors; mean NIHSS score was 6 (SD 7.5) without differences in relation to BAO subtype. The same was true for missing 24-h NIHSS scores. The rate of registered sICH was 1.1%. However, six of nine patients who died within 24 h had no postprocedural imaging, and none of the six patients without postprocedural imaging was registered with sICH.

**Table 3:** Early postprocedural outcomes and adverse events. Missing data: NIHSS 39.4%, sICH 27.1%

Variable	Proximal BAO *n* = 69 % (*n*)	Mid BAO *n* = 73 % (*n*)	Distal BAO *n* = 109 % (*n*)	All BAO *n* = 251 % (*n*)	*p* value
Early outcomes and adverse events					
24 h—NIHSS in survivors					
Median (IQR)	3 (2–14)	3 (2–14)	2 (1–8)	3 (1–8)	0.146
Mean (SD)	8 (9.0)	5 (7.5)	5 (6.5)	6 (7.5)	0.099
Death within 24 h	5.8% (4)	4.1% (3)	1.8% (2)	3.6% (9)	0.368
sICH	2.2% (1)	0% (0)	1.2% (1)	1.1% (2)	0.587
Any serious complication	49.3% (34)	21.9% (16)	23.9% (26)	30.3% (76)	< 0.001
Procedure-related	8.7% (6)	1.4% (1)	3.7% (4)	4.4% (11)	0.092
Postprocedural	43.5% (30)	20.5% (15)	22.0% (24)	27.5% (69)	0.002

*BAO* basilar artery occlusion, *NIHSS* National Institutes of Health Stroke Scale, *IQR* interquartile range, *SD* standard deviation, *sICH* symptomatic intracerebral haemorrhage

Serious complications occurred in 30.3% and were most frequent in proximal BAO (49.3%, middle 21.9%, distal 23.9%, *p* = 0.002, Table 3). The majority of complications were registered as postprocedural, see supplemental Table I a–d. Space-occupying ischaemic lesions were the most common postprocedural complication, occurring in 17 patients (of whom 10 had proximal BAO) and were fatal in 16/17 patients. Patients with this complication were characterized by severe symptoms at onset (median NIHSS score 30, half were comatose), by long process times and by more frequent use of angioplasty and/or stenting.

### Mortality

**Fig. 2:**
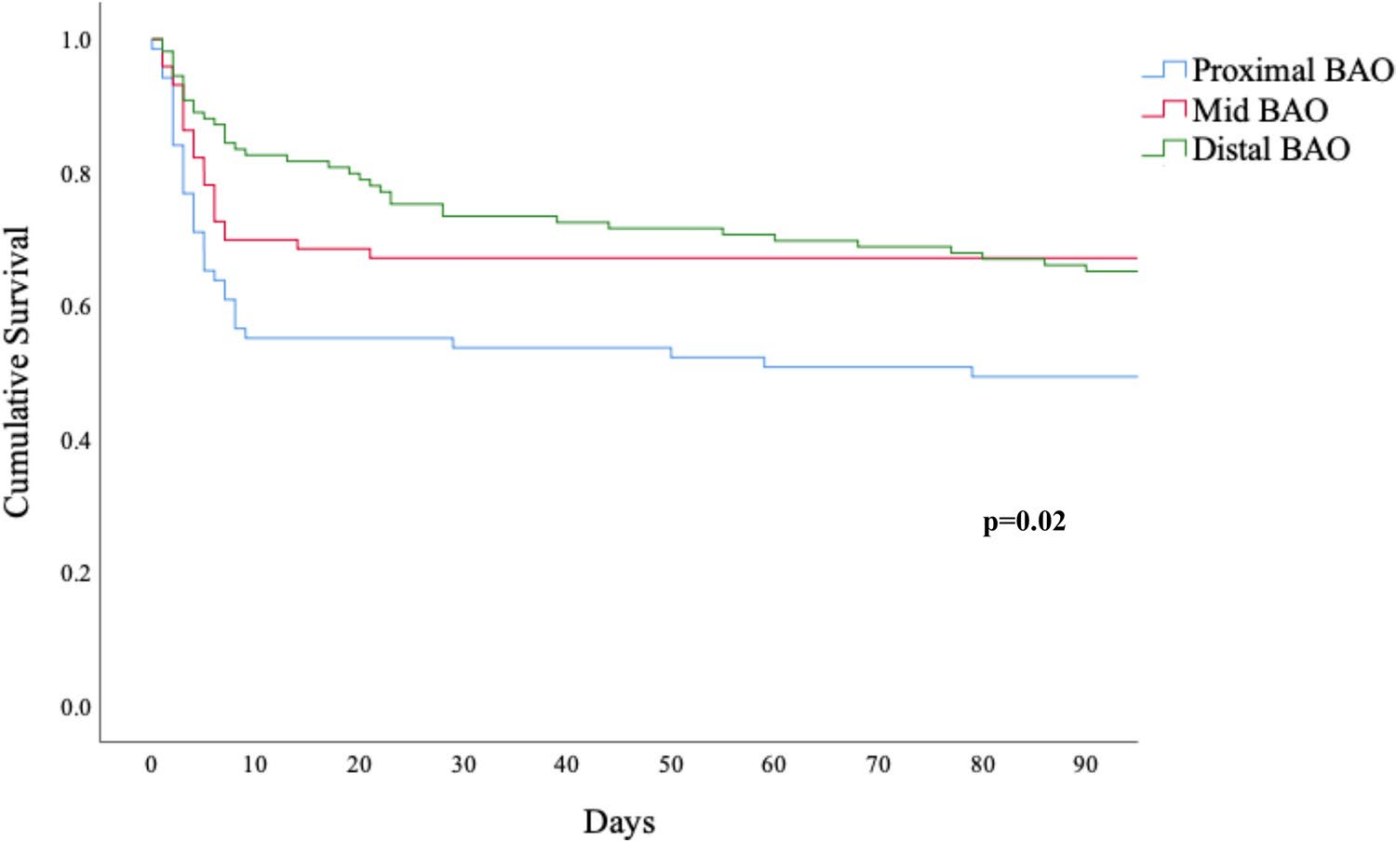
Ninety-day survival by basilar artery occlusion (BAO) subtype, log rank test *p* = 0.02

**Fig. 3:**
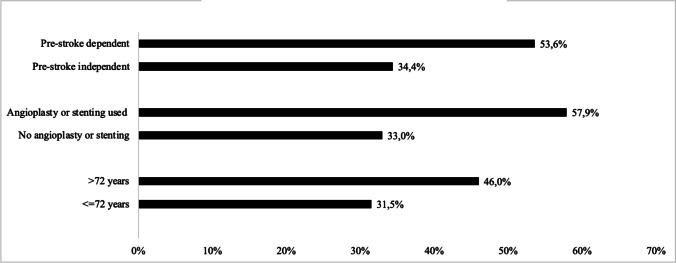
Case fatality at 90 days stratified by pre-stroke function (*n* = 28 dependents, 209 independents), use of angioplasty and/or stenting (*n* = 57, 194 without use of angioplasty/stenting) and by median age (72 years)

All-cause 90-day mortality was 38.6% (97/251), and survival differed significantly between BAO subtypes (*p* = 0.02, Fig. 2). Deaths within 28 days (33.5%, 84/251) accounted for most deaths (86.6%, 84/97) within 90 days and also differed by BAO subtype (proximal 44.9%, middle 32.9%, distal 26.6%, *p* = 0.041). Case fatality at 90 days stratified by median age (*p* = 0.003), pre-stroke function (*p* = 0.041) and use of angioplasty and/or stenting (*p* = 0.001) is shown in Fig. 3, and was higher in older and in pre-stroke dependent patients and in those where angioplasty and/or stenting was performed.

### Functional outcome

**Fig. 4:**
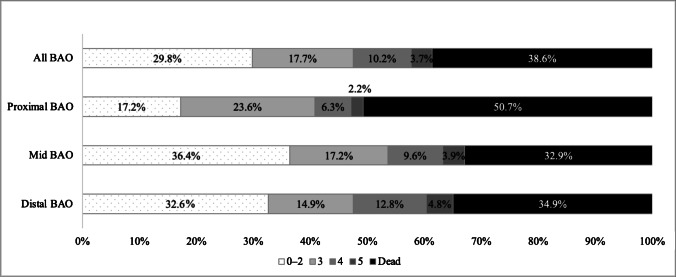
modified Rankin Scale distribution in 251 basilar artery occlusion (BAO) treated with endovascular therapy by occlusion location

There were 154 (61.4%) 90-day survivors of whom 114 were followed up and 40 (15.9%, 40/251) were lost to follow-up. Since data on death were complete, those lost to follow-up were alive but their functional status was unknown. Patients lost to follow-up were significantly younger and more often had an unknown time of onset, but apart from that, baseline characteristics and BAO subtype did not differ (supplemental Table II). mRS distribution by lesion location is shown in Fig. 4 and in supplemental Fig. I. Overall functional independence was 29.8% (21.9% when including lost to follow-up).

## Discussion

We present observational data on endovascular therapy for BAO from a large, nationwide, unselected 4-year cohort. A good outcome (mRS ≤ 2) at 90 days was observed in 29.8%, which is in line with the largest published observational studies and RCTs (27.4–35.1%) [13, 20, 27, 28]. All-cause mortality at 90 days was 38.6%, slightly higher than previously published data from comparable studies (29.9–36.8%) [13, 15, 20, 27, 28, 42], and considerably higher than recent studies using MRI-based patient selection (16–20.3%) [17, 24] in which patients with large brainstem infarcts were excluded. Swedish centres use CT in 98% of cases and likely treat patients who, given the higher sensitivity of MRI for early and infratentorial lesions, would have been excluded from MRI-based studies. This may partially explain the higher mortality rates in cohorts with CT-based patient selection, including ours. Moreover, some studies excluded pre-stroke dependent patients [13, 24, 25], while our material included 11.8% with pre-stroke dependency.

In our analyses, it is evident that patients with proximal BAO had higher mortality (50.7%) despite being 10 years younger than those with distal BAO. They were also more often male and smokers and fewer had atrial fibrillation than did patients with middle and distal BAO. One in seven patients with a proximal BAO died from a space-occupying ischaemic lesion, in part explaining the excess mortality in this group. In the BASILAR registry [13], mortality in patients with proximal BAO was 46.7%. One single-centre study [16] reported that 61.3% of the BAO patients with atherosclerotic stroke (50%) had a poor clinical outcome at 90 days and that proximal occlusions more often were associated with underlying stenosis. A recent multicentre study showed higher mortality in BAO stroke of atherosclerotic origin than other origins (38.8% vs 29.3%) [23]. Data on intracranial atherosclerotic disease (ICAD) in RSEVAS are incomplete, but stenting and angioplasty, which is strongly suggestive of underlying ICAD, were more commonly used in patients with proximal or middle BAO. Mortality in our angioplasty/stenting group was, irrespective of periprocedural antithrombotic treatment, considerably higher (57.9%) compared to vertebrobasilar artery occlusion treated with angioplasty/stenting in a previously published retrospective, multicentre study (33%) [43]. The indication for use of angioplasty/stenting was not registered in EVAS, but the most probable indication may have been a rescue strategy to achieve recanalization. One study described re-occlusion after initial reperfusion and need for rescue treatment with angioplasty or stenting in 63% of their posterior circulation ICAD patients [44].

Approximately 60% of BAO patients in Sweden were transferred from a primary hospital to a CSC, and these patients had a longer time to revascularization. Nearly half of the patients were drowsy or comatose at presentation, making it unsafe to triage patients directly to a CSC when a primary hospital is closer. Nevertheless, process times recorded in RSEVAS are shorter, including those for secondary transport patients, than recently published studies performed during the same time period [13, 21, 25].

General anaesthesia was used in nearly 80% of our patients, compared to 23.9–40.2% in recently published studies [13, 25]. The discrepancy may be explained by differences in clinical presentation and by local practice. Concerning treatment strategy, aspiration alone was used in 40.2% of BAO and stent retriever and aspiration in 54.5%, which differs from the BASILAR registry in which stent retrievers were used in 75% and aspiration alone only in 3.1% of patients. Nevertheless, the degree of successful revascularization (mTICI 2b–3) was comparable to the BASILAR registry.

The proportion of sICH registered in RSEVAS (1.1%) is much lower compared to 7.1% in the BASILAR registry [13]. It is probable that sICH is underestimated in RSEVAS, since six of nine patients who died within 24 h did not have follow-up stroke imaging, and any haemorrhagic cause of death would remain undetected in these patients.

### Strengths

RSEVAS is an unselected nationwide database, containing real-world data with high coverage, and reflecting current clinical practice and outcome.

### Limitations

This study has several limitations: (1) loss to follow-up was 15.9%, and patients lost to follow-up were significantly younger. This may lead to attrition bias in terms of underestimating dependency levels. We chose to present data both including (supplemental Fig. I) and omitting those lost to follow-up from analyses but did not use multiple imputation for missing data. (2) RSEVAS catches > 90% of all EVTs performed in Sweden, but data on BAO patients not receiving EVT are not available in any register. (3) Data on functional outcome are self-reported, which may lead to both over- and underestimation of own abilities. However, self-reported data have shown good agreement to objectively assessed mRS at 90 days [39]. (4) No core lab imaging assessment was used, which may have affected results, probably in a more positive direction [45]. (5) Data collection in EVAS concerning collateral circulation and ICAD was not reliable during the study period, and is therefore not specifically mentioned, although both are important prognostic factors [20, 46]. Data on underlying dissection were incomplete and therefore not used. (6) Probable stroke mechanism was not available in either register. (7) Occlusions were defined by their proximal end, meaning that some proximal and middle occlusions may have involved more distal segments. However, neither NIHSS nor LOC differed by occlusion location, and 61.8% of proximal BAO patients were alert on admission, indicating that the majority of proximal occlusions did not extend to more distal segments. (8) NIHSS, especially at 24 h, may be underestimated due to missing data. (9) sICH may be underestimated due to missing data.

## Conclusions

We confirm a serious outcome in BAO despite endovascular therapies, and demonstrate important differences relating to occlusion location in baseline characteristics, procedural time, therapeutic measures and outcome. Further in-depth analyses of factors affecting outcome in BAO are warranted.

## Supplementary Information

Below is the link to the electronic supplementary material.

Supplementary file1 (PDF 415 KB)

Supplementary file2 (DOCX 247 KB)

## Data Availability

Requests to access an anonymized dataset or code supporting the conclusions of this article may be sent to RS after obtaining the appropriate ethics approval.
