# Supplementary material for: Endovascular therapy in basilar artery occlusion in Sweden 2016–2019—a nationwide, prospective registry study
Source: Neuroradiology. 2021 Oct 30;64(5):959–68. doi: 10.1007/s00234-021-02843-3 (PMC9005406; doi:10.1007/s00234-021-02843-3)
Supplement: Supplementary file 2 — Supplementary file2 (DOCX 247 KB) [file 234_2021_2843_MOESM2_ESM.docx]

# Supplemental appendix

Supplemental Table I a. Procedure-related serious complications.

| **I a. Procedure-related complications** | **Proximal BAO n=69**  **n** | **Mid BAO**  **n=73**  **n** | **Distal BAO n=109**  **n** |
| --- | --- | --- | --- |
| Symptoms of hypoperfusion |  |  |  |
| Anesthesiologic problems |  |  |  |
| Related to endovascular device | 1 |  |  |
| Other |  |  |  |
| Vessel perforation |  |  |  |
| Extradural |  |  |  |
| Intradural | 3 |  |  |
| Perforator damage |  | 1 |  |
| Serious cardiac arrhythmia |  |  |  |
| Thromboembolic complication in lower extremities | 1 |  |  |
| Complications caused by defect devices |  |  |  |
| Groin problems after puncture that require treatment |  |  | 1 |
| Extracranial dissection | 1 |  | 2 |
| Other |  |  | 1 |
| **Total** | **8.7% (6/69)** | **1.4% (1/73)** | **3.7% (4/109)** |

Supplemental Table I b. Postprocedural serious complications.

| **I b. Postprocedural complications affecting recovery** | **Proximal BAO n=69**  % (n) | **Mid BAO n=73**  % (n) | **Distal BAO n=109**  % (n) | **All**  **N=251**  % (n) |
| --- | --- | --- | --- | --- |
| Pneumonia | 13% (9) | 6.8% (5) | 5.5% (6) | 8% (20) |
| Pulmonary edema | 0% (0) | 1.4% (1) | 1.8% (2) | 1.2% (3) |
| Arrhythmia | 1.4% (1) | 0% (0) | 1.8% (2) | 1.2% (3) |
| Myocardial infarct | 0% (0) | 0% (0) | 0.9% (1) | 0.4% (1) |
| Epileptic seizures | 0% (0) | 0% (0) | 1.8% (2) | 0.8% (2) |
| Urosepsis | 0% (0) | 0% (0) | 0% (0) | 0% (0) |
| Urinary tract infection | 0% (0) | 1.4% (1) | 0.9% (1) | 0.8% (2) |
| Other infection | 1.4% (1) | 0% (0) | 0.9% (1) | 0.8% (2) |
| Delirium | 1.4% (1) | 0% (0) | 0% (0) | 0.4% (1) |
| Intracranial hypertension /Space-occupying infarcts | 14.5% (10) | 6.8% (5) | 1.8% (2) | 6.8% (17) |
| Locked-in state | 1.4% (1) | 1.4% (1) | 0% (0) | 0.8% (2) |
| Re-occlusion | 5.8% (4) | 0% (0) | 0.9% (1) | 0.8% (5) |
| New EVT | 2/4 | 0 | 1/1 | 3/5 |
| Kidney failure | 0% (0) | 0% (0) | 0% (0) | 0% (0) |
| Fall accident | 0% (0) | 1.4% (1) | 0.9% (1) | 0.8% (2) |
| Groin problems that require treatment | 0% (0) | 0% (0) | 0.9% (1) | 0.4% (1) |
| Other | 4.3% (3) | 1.4% (1) | 3.7% (4) | 3.2% (8) |
| **Total** | **43.5% (30)** | **20.5% (15)** | **22% (24)** | **27.5% (69)** |

Supplemental Table I c. Proportion of patients who died within seven days by type of complication and occlusion location. Number of complications retrieved from Table I a and b above.

| **Procedure-related complications associated with death** | **Proximal BAO complications**  Dead % (n/N) | **Mid BAO**  **complications**  Dead % (n/N) | **Distal BAO**  **complications**  Dead % (n/N) | **All**  **complications**  Dead % (n/N) |
| --- | --- | --- | --- | --- |
| Hypoperfusion | 0% (0/1) | 0% (0) | 0% (0) | 0% (0/1) |
| Intradural vessel perforation | 33% (1/3) | 0% (0) | 0% (0) | 33% (1/3) |
| Perforator damage | 0% (0) | 100% (1/1) | 0% (0) | 100% (1/1) |
| Thromboembolic complication in lower extremities | 0% (0/1) | 0% (0) | 0% (0) | 0% (0/1) |
| Groin problems after puncture that require treatment | 0% (0) | 0% (0) | 100% (1/1) | 100% (1/1) |
| Extracranial dissection | 100% (1/1) | 0% (0) | 100% (2/2) | 100% (3/3) |
| Other | 0% (0) | 0% (0) | 100% (1/1) | 100% (1/1) |
| **Total** | **33.3% (2/6)** | **100% (1/1)** | **25% (1/4)** | **36.3% (4/11)** |
| **I c. Postprocedural complications associated with death** | **Proximal BAO complications**  Dead % (n/N) | **Mid BAO**  **complications**  Dead % (n/N) | **Distal BAO**  **complications**  Dead % (n/N) | **All**  **complications**  Dead % (n/N) |
| Pneumonia | 11.1% (1/9) | 40% (2/5) | 0% (0/6) | 15% (3/20) |
| Pulmonary edema | 0% (0) | 0% (0/1) | 0% (0/2) | 0% (0/3) |
| Arrhythmia | 0% (0/1) | 0% (0) | 50% (1/2) | 33.3% (1/3) |
| Myocardial infarct | 0% (0) | 0% (0) | 100% (1/1) | 100% (1/1) |
| Epileptic seizures | 0% (0) | 0% (0) | 0% (0/2) | 0% (0/2) |
| Urosepsis | 0% (0) | 0% (0) | 0% (0) | 0% (0) |
| Urinary tract infection | 0% (0) | 0% (0/1) | 0% (0/1) | 0% (0/2) |
| Other infection | 0% (0/1) | 0% (0) | 0% (0/1) | 0% (0/2) |
| Delirium | 0% (0/1) | 0% (0) | 0% (0) | 0% (0/1) |
| Intracranial hypertension /Space-occupying infarcts | 100% (10/10) | 80% (4/5) | 100% (2/2) | 94.1% (16/17) |
| Locked-in state | 0% (0/1) | 100% (1/1) | 0% (0) | 50% (1/2) |
| Re-occlusion | 25% (1/4) | 0% (0) | 0% (0/1) | 20% (1/5) |
| Kidney failure | 0% (0) | 0% (0) | 0% (0) | 0% (0) |
| Fall accident | 0% (0) | 100% (1/1) | 0% (0/1) | 50% (1/2) |
| Groin problems that require treatment | 0% (0) | 0% (0) | 0% (0/1) | 0% (0/1) |
| Other | 33.3% (1/3) | 0% (0/1) | 0% (0/4) | 12.5% (1/8) |
| **Total** | **43.3% (13/30)** | **53.3% (8/15)** | **16.7% (4/24)** | **36.2% (25/69)** |

Supplemental Table I d. Selected characteristics in 16 patients who died from intracranial hypertension/expansive infarcts within 7 days. Missing data: hypertension, diabetes, AF, previous stroke, LOC (n=1), IVT (n=2), NIHSS (n=4)

| **Variable** | **Died < 7 days**  **n=16**  **% (n)** |
| --- | --- |
| **Demographic characteristics** |  |
| Median age (IQR)* | 63.5 (57–73) |
| Female sex | 37.5% (6) |
| **Pre-stroke function** |  |
| mRS 0–2 | 100% (16) |
| **Vascular risk factors** |  |
| Hypertension | 43.8% (7) |
| AF | 12.5% (2) |
| Diabetes | 0% (0) |
| Previous stroke | 18.8% (3) |
| **Clinical characteristics** |  |
| NIHSS score (IQR) | 30 (11­–30) |
| Level of consciousness |  |
| alert | 18.8% (3) |
| drowsy | 25% (4) |
| comatose | 50% (8) |
| **Treatment** |  |
| IVT | 50% (8) |
| **BAO location** |  |
| Proximal | 62.5% (10) |
| Mid | 25% (4) |
| Distal | 12.5% (2) |
| **Pre-EVT brain imaging findings** |  |
| Any infarct finding | 37.5% (6) |
| Cerebellar infarct | 31.3% (5) |
| Brainstem infarct | 12.5% (2) |
| **Therapy and median process time** |  |
| OTR (IQR) | 514 (357–901) |
| Groin to revascularization (IQR) | 66 (32–130) |
| Use of angioplasty or stenting | 43.8% (7) |
| Successful recanalization (mTICI 2b-3) | 62.5% (10) |
| **Post-EVT brain imaging findings at 24 hours** | |
| Any infarct finding | 87.5% (14) |
| Cerebellar infarct | 87.5% (14) |
| Brainstem infarct | 75% (12) |

Supplemental Table II. Characteristics in followed up vs. not followed up subjects.

| **Variable** | **Followed up**  **n=211**  **% (n)** | **Lost to follow-up**  **n=40**  **% (n)** |
| --- | --- | --- |
| **Demographic characteristics** |  |  |
| Median age (IQR)* | 73 (62–80) | 66 (54–75) |
| Female sex | 42.7% (90) | 40.0% (16) |
| Single household | 34.1% (72) | 32.5% (13) |
| **Pre-stroke function** |  |  |
| mRS 0–2 | 87.5% (175) | 91.9% (34) |
| mRS 3–5 | 12.5% (25) | 8.1% (3) |
| **Vascular risk factors** |  |  |
| Hypertension | 64.5% (136) | 55.0% (22) |
| AF | 32.4% (68) | 30.0% (12) |
| Diabetes | 20.1% (42) | 20.0% (8) |
| Current smoking | 16.7% (27) | 27.3% (9) |
| Previous stroke | 15.6% (33) | 7.5% (3) |
| Previous TIA | 5.7% (12) | 0% (0) |
| **Clinical characteristics** |  |  |
| NIHSS score (IQR) | 14 (7–30) | 14 (5–25) |
| Level of consciousness |  |  |
| alert | 50.0% (105) | 64.1% (25) |
| drowsy | 23.3% (49) | 15.4% (6) |
| comatose | 20.5% (56) | 20.5% (8) |
| **Treatments** |  |  |
| Ongoing anticoagulation* | 14.7% (31) | 5.0% (2) |
| IVT | 34.3% (69) | 35.0% (14) |
| OTN time (min, IQR) | 124 (101–180) | 156 (112–218) |
| DTN time (min, IQR) | 69 (39–88) | 69 (24–101) |
| **Time of onset** |  |  |
| Known or estimated* | 86.3% (182) | 67.5% (27) |
| **BAO location** |  |  |
| Proximal | 27.5% (58) | 27.5% (11) |
| Mid | 28.0% (59) | 35.0% (14) |
| Distal | 44.5% (94) | 37.5% (15) |
| *p-value <0.05, BAO=basilar artery occlusion, IQR=interquartile range, OTN= Onset to needle, DTN= Door to needle, IVT=intravenous thrombolysis | | |


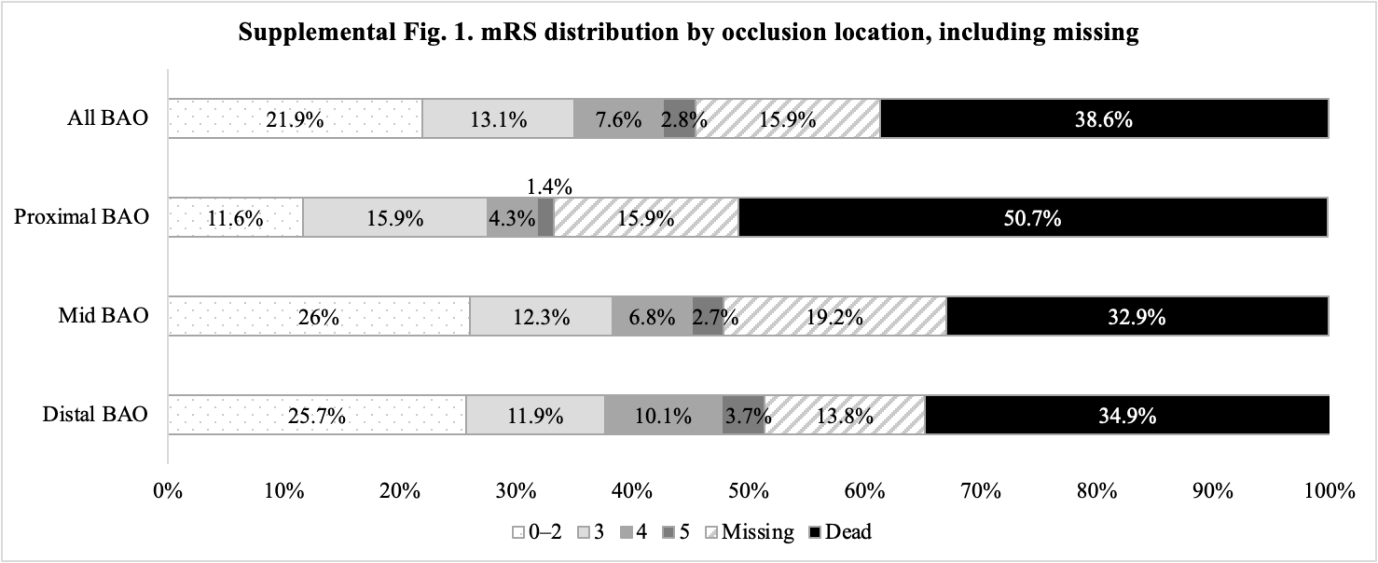


Supplemental Fig. I. mRS distribution at 90 days in all patients including missing (n=40) as a separate category.
